# Viral Protein VP1 Virus-like Particles (VLP) of CVB4 Induces Protective Immunity against Lethal Challenges with Diabetogenic E2 and Wild Type JBV Strains in Mice Model

**DOI:** 10.3390/v15040878

**Published:** 2023-03-29

**Authors:** Jawhar Gharbi, Ikbel Hadj Hassine, Mouna Hassine, Mohammed Al-Malki, Ameera Al-Yami, Anwar Al-Bachir, Manel Ben M’hadheb

**Affiliations:** 1 Department of Biological Sciences, College of Science, King Faisal University, P.O. Box 380, Al-Ahsa 31982, Saudi Arabia; 2 Research Unit UR17ES30 «Virology & Antiviral Strategies», Institute of Biotechnology, University of Monastir, Monastir 5000, Tunisia

**Keywords:** coxsackievirus B4, virus-like particles, viral protein VP1, vaccine, lethal challenge, immunization, type 1 diabetes

## Abstract

Several epidemiological studies demonstrated that coxsackievirus B4 (CVB4) causes viral pancreatitis and can ultimately result in type 1 diabetes mellitus (T1D). Prevention of CVB4 infection is therefore highly desirable. There is currently no vaccine or antiviral therapeutic reagent in clinical use. VLP are structurally similar to native virus particles and therefore are far better immunogens than any other subunit vaccines. Many studies have shown the potential of capsid protein VP1 on providing protective effects from different viral strains. In this study, we contributed towards the development of a CVB4 VLP-based vaccine from the total protein VP1 of the diabetogenic CVB4E2 strain and assessed whether it could induce a protective immunity against both the wild-type CVB4JBV and the diabetogenic CVB4E2 strains in mice model. Serum samples, taken from mice immunized with VLP, were assayed in vitro for their anti-CVB4 neutralizing activity and in vivo for protective activity. We show that VLP vaccine generates robust immune responses that protect mice from lethal challenges. Results demonstrate that CVB4 VP1 capsid proteins expressed in insect cells have the intrinsic capacity to assemble into non-infectious VLP, which afforded protection from CVB4 infection to mice when used as a vaccine.

## 1. Introduction

Type 1 diabetes (T1D), initially termed insulin-dependent diabetes mellitus (IDDM), is an organ-specific auto-immune disease characterized by a defect in insulin production, as a result of selective and massive destruction of islets β cells (80 to 90%) or of impairment of their functions. The progression of the auto-immune process is generally slow and may take several years before the onset of the disease. Starting generally at a young age, T1D is also qualified as juvenile. Indeed, it concerns especially children and young adults and occurs generally before the age of 40 years with incidence peaks at 2, 4–6, and 10–14 years [1]. T1D represents 5 to 10% of all cases of diabetes, a disease with frequently grave and heavy consequences for the patient (e.g., ketoacidosis, retinopathies, hypertension, and ischemic cardiopathy). Polyuria, polyphagia, polydypsia, weight loss, weakness, and recurrent infections are the principal signs and symptoms. The annual incidence of T1D being rather variable from one country to another (from less than 1 per 100,000 habitants in Asia to approximately 14 per 100,000 in USA, and exceeding 30 per 100,000 in Scandinavia) is, however, in continual increase all over the world (approximately 3% per year between 1960 and 2000), especially among children less than five years old [2,3].

T1D is a multifactorial disease that obeys a genetic predisposition, determined by a balance between susceptibility and resistance alleles, and is triggered by environmental factors. Environmental factors, especially viruses, are thought to play an important role in the initiation or the acceleration of T1D pathogenesis. Sero-epidemiological data largely implicated enteroviruses, especially type B coxsackieviruses (CVB), in T1D development [4,5,6,7]. The isolation of CVB4 from the pancreas of diabetic patients strengthened the hypothesis of a relationship between the virus and the disease. Furthermore, studies performed in vitro and in vivo in animal models resulted in a better knowledge of the role of CVB4 in T1D. These studies helped in discovering pathogenic mechanisms of the infection that can lead to β cell destruction.

Enteroviruses belonging to the *Enterovirus* genus and the *Picornaviridae* family have a ubiquitous distribution and are mainly transmitted through the fecal–oral route by ingestion of food or water soiled by contaminated feces. Coxsackievirus B4 (CVB4), one of the six CVB serotypes and a member of the Enterovirus B species, is characterized by non-enveloped icosahedral virions of 25–30 nm in diameter and a 7.4 kb positive-sense RNA with a single long open reading frame consisting of three regions P1–P3. The P1 region encodes the four viral shell proteins: viral protein-0 (VP0) (cleaved to give VP4 and VP2 during viral maturation), VP3, and VP1, whereas the P2 and P3 regions encode the seven nonstructural proteins including the 3CD protease that cleaves P1 into the four structural proteins. The icosahedral capsids of coxsackieviruses are composed of 60 identical units formed by VP1, VP2, VP3, and VP4. VP1 is the major antigen since it can provoke the immune system easily by its antigenic site on the virus surface. Furthermore, anti-picornavirus drugs and neutralizing antibodies can easily bind to VP1, more precisely to its hydrophobic tunnel causing conformational changes that may block the attachment to host cells and consequently preventing the infection [8]. Several investigators have found enteroviral RNA in peripheral blood of 27 to 64% of patients with T1D [3]. In some cases, CVB RNA sequences showed a significant homology with CVB4 [4]. In another study, sequences of CVB4 RNA homologous to coxsackievirus B4E2 (CVB4E2) were found in peripheral blood mononuclear cells of patients with T1D [9]. CVB4E2 is the most known diabetogenic enterovirus strain. It was isolated from the pancreas of a child who died from diabetic ketoacidosis and was able to destroy β cells of the pancreas and to induce hyperglycemia in some susceptible mouse strains [10]. A persistent infection of human pancreatic islet cells in vitro can be obtained with CVB4E2 [11]. The assumption that enteroviruses cause β cell cytolysis is supported by observations describing the presence of viral antigens in human β cells as well as the destruction of islet cells in patients deceased from systemic enteroviruses [12]. The presence of enterovirus RNA and/or of infectious virus, especially CVB4 in the pancreatic islets in T1D subjects, was also reported [13]. Other researchers described the detection of enteroviral VP1 in post-mortem pancreatic tissue of a child who was tested positive for islet-cell autoantibodies, before developing diabetes and without any inflammatory changes in the islets, which is in agreement with a possible role of enteroviruses in the early stages of the pathogenic process [14]. The diabetogenic strain CVB4E2 was able to induce hyperglycemia in some susceptible mouse strains [10]. Mice constitute a universal model for studying several aspects related to T1D that are difficult to investigate in humans. It has been observed that the virus can infect the pancreatic islet cells in a CVB4E2-induced T1D in mice model, which resulted in the development of T1D.

Prevention of T1D remains a challenge in part because its exact cause is still unknown. In fact, there is not any factor retained as the unique cause and T1D seems rather to be the resultant of interactions of a multitude of factors. Although vaccination against CVB4 could reduce the incidence of this chronic auto-immune disease, there is currently no therapeutic reagent or vaccine in clinical use. Recently, many vaccine models against CVB using different strategies were developed to prevent T1D [15,16,17]. Viral capsid protein 1 (VP1) is one of the major immunogenic capsid proteins of the enteroviruses genus. Several studies have shown the potential of VP1 in both the diagnosis and vaccine development against picornaviruses [18,19]. In this study, we contributed towards the development of a CVB4 vaccine by producing the viral capsid protein VP1 virus-like particles (VP1-VLP) and we evaluated their immunogenicity in administered intraperitoneally mouse model for coxsackievirus.

## 2. Materials and Methods

### 2.1. Viruses, Cells and Media

The diabetogenic strain CVB4E2 (provided by J.W. Yoon, Julia Mc Furlane Diabetes Research Center, Alberta, Canada) isolated in 1979 from the pancreas of a child who died from diabetic ketoacidosis [10] and the wild-type strain CVB4JBV were propagated in HeLa cells (human cervical carcinoma cells) in Eagle’s minimal essential medium (MEM) supplemented with 10% heat-inactivated fetal calf serum (FCS) (Sigma, St. Louis, MO, USA), 1% L-glutamine, 50 µg/mL of streptomycin, 50 UI/mL of penicillin (Bio Whittaker, Walkersville, MD, USA), 1% nonessential amino acids (Gibco BRL, Grand Island, NE, USA), and 0.05% Fongizone (Amphotericin B). Supernatants were collected 3 days after inoculation, clarified at 2000× *g* for 10 min, divided into aliquots, and stored at −80 °C. Virus titers in stocks were determined in HeLa cells by limiting dilution assays for 50% tissue culture infectious doses (TCID_50_) according to Reed–Muench method. Monolayer of *Trichoplusiani* (High Five) cells (Gibco BRL) were maintained in suspension at mid-log phase and grown at 27 °C in TC-100 Insect Medium (Sigma) supplemented with 10% FCS (Sigma), 10,000 UI/mL Penicillin, 50 mg/mL Gentamicin, 0.25 mg/mL Fungizone, and 50 mg/mL Antimiotic. The cells were grown in cell culture dish 150 × 20 mm and sub-cultured every 3–4 days.

### 2.2. Design and Preparation of the VP1-VLP Vaccine

The VP1-VLP (virus-like particles) consisting of the total viral capsid protein VP1 of the diabetogenic CVB4E2 strain was produced using the Bac-to-Bac vector system from recombinant baculovirus in infected insect cells as previously described [20]. Briefly, pFastBac1 (Invitrogen) was used as a backbone and the gene coding for VP1 capsid protein, previously cloned in an expression vector, was extracted and subcloned into the corresponding sites (*Bam*HI/*Hin*dIII) under the Polyhedrin promoter (PpH). The expression cassette limited by Tn7R and Tn7L was then transferred into the bacmid by site-specific transposition. To obtain VLPs, High Five cells’ monolayers were co-infected with purified recombinant baculoviruses expressing recombinant VP1 capsid protein (rVP1) and cultured in TC-100 insect medium without FCS at 27 °C. Culture supernatants were collected by centrifugation at 5000× *g* for 20 min to remove cells. The collected supernatants containing VP1-VLP were further spun by ultracentrifugation at 170,000× *g* for 150 min. The VP1-VLP pellets were resuspended in phosphate-buffered saline (PBS) and VP1-VLP were further purified by ultracentrifugation with discontinuous 25%–35%–60% sucrose gradient at 200,000× *g* for 45 min at 4 °C [14]. The particulate material containing the VLPs was collected in different fractions from top to bottom and the sample of each was mixed with the buffer, pelleted finally (240,000× *g* for 120 min at 4 °C) in an SW55 Ti Swinging-Bucket Rotor (Backman Coulter, Brea, CA, USA), and resuspended in 100 µL PBS Buffer. Produced VLPs were then analyzed by transmission electron microscopy.

### 2.3. Mouse Immunization, Blood/Tissues Collection and Challenge Schedule

The King Faisal University Ethical Committee approved our study protocol. The animal care and use protocol adhered to the regulations of the Institutional Committee (DSR). Five-week-old female *Swiss Albinos* mice were divided randomly into six groups of five mice each (*n* = 5). Group 1 mice were intraperitoneally (ip) prime inoculated at day 0 and boosted at day 15 with 0.5 µg of VP1-VLP vaccine. Group 2 mice received a prime-boost regimen at days 0 and 15 and challenged at day 22 with 1.1 × 10^6^ TCID_50_ of CVB4E2 strain. Group 3 mice received a prime-boost regimen at days 0 and 15 and challenged at day 22 with 1.36 × 10^6^ TCID_50_ of CVB4JBV strain. Groups 4 and Group 5 mice received phosphate buffered saline (PBS) at days 0 and 15 and challenged at day 22, respectively, with CVB4E2 and CVB4JBV strains. Group 6 mice represented the naive control group. All animals were monitored daily for clinical signs and body weight changes, and the occurrence of mortality served as the experimental endpoint.

**Figure 1:**
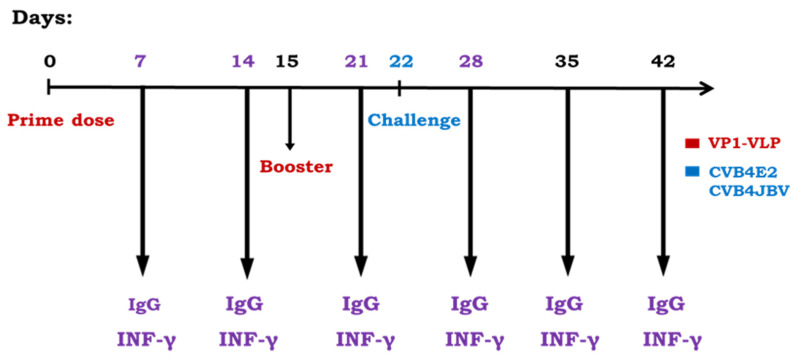
Mice immunization and challenge schedule. Mice Group 1 were intraperitoneally (ip) immunized with a prime-boost regimen at days 0 and 15. Immunized mice Group 2 and Group 3 were immunized with a prime-boost regimen and then challenged at day 22 successively with CVB4E2 and CVB4JBV strains. Mice Group 4 and Group 5 received PBS at days 0 and 15 and challenged successively with CVB4E2 and CVB4JBV strains. Group 6 mice represent the naive control group receiving only PBS at days 0 and 15. Neutralizing antibody and cytokine titers were measured in serum collected from mice tails at days 7, 14, 21, 28, 35, and 42.

Mice were treated according to general ethic rules and maintained under specific pathogen-free conditions with unlimited access to food and water. All animals were sacrificed 20 days after lethal challenges (42 days post first immunization). Portions of pancreas tissues were removed, rinsed with PBS, snap-frozen in liquid nitrogen, and stored at −80 °C for virus titrations. Bloods were collected from the mice tails on days 7, 14, 21, 28, 35, and 42 post prime inoculation for specific antibody assays and for cytokine interferon INF-γ detection (Figure 1).

### 2.4. Micro-Neutralization Assay and Determination of Neutralizing Antibody Titers

Suspended HeLa cells were first seeded at 8 × 10^3^ cells per well in 96-well microtiter plates (Falcon). After 16 h of incubation at 37 °C in a humidified incubator with 5% CO_2_, 50 µL of MEM supplemented with 2% FCS, 1% L-glutamine, 50 µg/mL streptomycin, 50 UI/mL penicillin, 1% non-essential amino acids, and 0.05% fungizone was added in each well. Serum samples collected from immunized mice were inactivated for 30 min at 56 °C. Neutralizing antibodies titers were determined using a micro-neutralization assay with HeLa cells. Briefly, 50 µL of serial serum dilutions (two-fold gradient diluted from 1:10 to 1:5120) was mixed, respectively, with 50 μL of 100 TCID_50_ CVB4E2 or CVB4JBV in a 96-well plate, and HeLa cell suspensions (final concentration 8 × 10^3^ cells) were added 2 h later. After incubation for 6 days at 37 °C, neutralizing antibodies were determined as the highest dilutions of serum that totally inhibited the viral cytopathic effect (CPE).

### 2.5. VLP-VP1-Specific IgG Antibody Titers and IgG Subtypes Distribution

Serum IgG antibodies were assessed using ELISA method. The 96-well plates were coated with 0.2 µg/well of purified VP1-VLP and blocked with 3% bovine serum albumin (BSA) in PBS for 2 h at room temperature to prevent non-specific binding. Plates were then washed three times with PBS + 0.1% Tween-20 and blocked with 3% BSA (100 µL/well) for 2 h at room temperature. Serum samples were thawed on ice and serially diluted 10 times in PBS starting at 1:10 dilution. Plates were then washed with PBS + 0.5% Tween-20 and 100 μL serial diluted sera in PBS was added to each well and incubated for 4 h at 37 °C. After washing with PBS, the HRP (horseradish peroxidase)-conjugated goat anti-mouse IgG secondary antibody (BD Biosciences, San Jose, CA, USA) was added to wells (50 µL/well) at a dilution of 1:4000. The plates were then incubated at 37 °C for 1 h. Finally, after being washed with PBS and added 50 µL/well with TMB (3, 3’,5, 5’-Tetramethylbenzidine, BD Biosciences), the reaction was stopped by adding 50 µL/well of 0.3 M sulfuric acid (Sigma) and measurements were taken at 450 nm by an absorbance microplate reader plate. The cut-off value (COV) of the OD was established as the average + 2 standard deviations of serum from control mice. For a given sample in each group, the highest dilution of sera that had an OD above the COV was considered as a positive titer. For each day post immunization, the antibody titers were averaged to obtain the geometric mean titer and compared between groups.

The same method of ELISA was performed to determine IgG antibody subtypes in the mouse sera. Purified VP1-VLP was used as antigen and serial dilutions of mice sera in BSA were added as different subtypes of antibodies. Conjugate antibodies goat anti-mouse IgG1-HRP, goat anti-mouse IgG2a-HRP, goat anti-mouse IgG2b-HRP, and goat anti-mouse IgG3-HRP were used to detect successively IgG antibody subtypes IgG1, IgG2a, IgG2b, and IgG3.

### 2.6. Cytokine Detection and Concentration Determination

The measurement of interferon gamma (IFN-γ) was conducted to demonstrate whether VP1-VLP vaccine could elicit the cellular immunity. Sera from VP1-VLP immunized and challenged mice groups and from naive control mice group were collected at days 7, 14, 21, 28, 35, and 42 after the prime immunization. The concentrations of IFN-γ were determined and evaluated by ELISA method, using the Mouse IFN-γ platinum ELISA kit (Thermo Fisher, Waltham, MA, USA).

### 2.7. Tissues Collection and Viral Titration

Pancreatic tissues were isolated from mice 20 days after challenge (42 days post first immunization). Snap-frozen tissues were crushed using a tissue ruptor (Qiagen, Hilden, Germany), then centrifugated at 2000 rpm for 10 min at 4 °C and homogenated in PBS at 1% antibiotics (penicillin and streptomycin) and stored at −80 °C for analysis. HeLa cells were used as a propagation system for growth of the CVB4 strains. The HeLa cells were seeded at 1 × 10^6^ on a six-well plate in Eagle’s Minimum Essential Medium (MEM) supplemented with 10% FCS and left for 24 to 48 h until 90% confluence. Supernatants were inoculated to HeLa cells. Cultures were then daily examined for the enterovirus cytopathic effect until day 7 post infection. The titers of infectious virus particles were expressed as TCID_50_ values, calculated by the method of Reed and Muench [21]. Blind passages were systematically performed for negative samples.

## 3. Results

### 3.1. Construction and Characterization of VP1-VLP Vaccine

**Figure 2:**
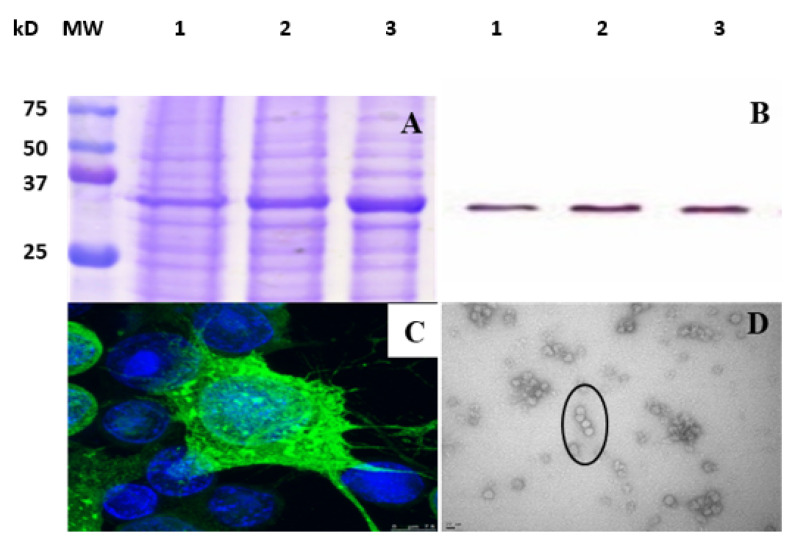
Characterization of the ultracentrifugation-purified VP1-VLP vaccine particles. (**A**) SDS-PAGE analysis of the three different fractions (Lanes 1–3) of ultracentrifugation-purified VP1-VLP. MW: molecular weight marker (10–250 kD). (**B**) Western blot analysis of purified VP1-VLP revealed that the VP1-VLP were recognized by an anti-enterovirus VP1 5D-8/1 clone monoclonal antibody (mAb). Lanes 1–3: fractions 1–3 of ultracentrifugation-purified VP1-VLP stock produced by Bac-VP1. (**C**) Immunofluorescence (IF) microscopy photo analysis of High Five infected cells at 2 days post infection revealed the presence of VP1-VLP particles in the cytoplasm of cells stained (fluorescence color) by the 5-D8/1 mAb with the goat anti-mouse coupled to AlexaFluor 488. Cell nuclei were stained with DAPI (blue color). Zoom 3X STED. (**D**) Transmission electron micrographs (TEM) of ultracentrifugation-purified VLPs. Scale bars, 20 nm.

The VP1-VLP (virus-like particles) consisting of the total viral capsid protein VP1 of diabetogenic CVB4E2 strain was produced using the Bac-to-Bac vector system from recombinant baculovirus in infected High Five insect cells. The expression of purified VP1-VLP was verified in supernatant by immunofluorescence staining, Western blot, and SDS-PAGE, and then characterized by transmission electron microscopy (TEM). SDS-PAGE results showed that the molecular weights matched with the theoretical prediction, 33 kD (Figure 2A). Moreover, the produced particles were recognized by the mouse anti-enterovirus VP1 5D-8/1 clone monoclonal antibody (Figure 2B,C). Produced particles demonstrated that they resembled the CVB4 particles in morphology and size by retaining their icosahedral symmetry with a diameter approximately of 25 nm as seen in Figure 2D.

### 3.2. Specific Neutralizing Antibodies Anti-E2 and Anti-JBV Responses

**Figure 3:**
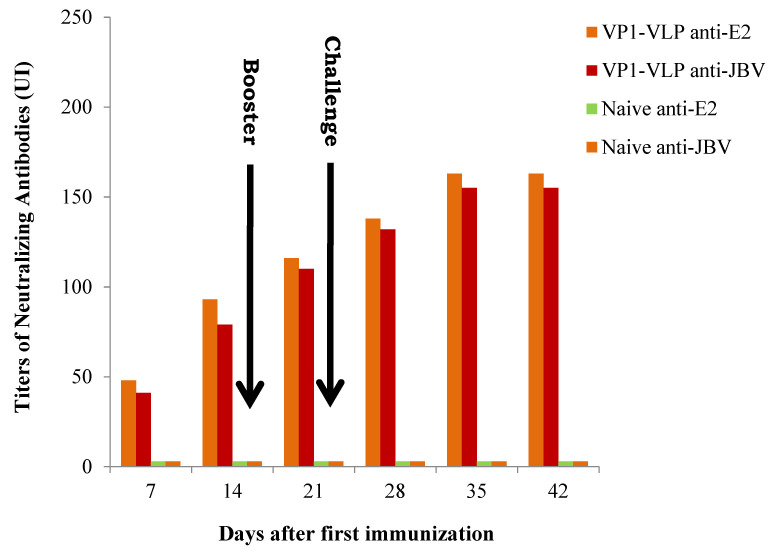
Production and titers of specific neutralizing antibodies in mice vaccinated with VP1-VLP. VP1-VLP anti-E2 represents mice group vaccinated with a prime-boost regimen at days 0 and 15, then challenged at day 22 with CVB4E2 strain. VP1-VLP anti-JBV represents mice group vaccinated with a prime-boost regimen at days 0 and 15, then challenged at day 22 with CVB4JBV strain. Titers of neutralizing antibodies produced in mice sera were measured at different times post immunization. Naïve control mice represent the negative control.

The efficacy of the VP1-VLP vaccine was assessed by its administration to mice groups. The purified vaccine particles VLP-VP1 were used to immunize mice at day 0 by intraperitoneally (ip) injections as described in Section 2. The animals were boosted one time at day 15 (Figure 1), and the antibody titer changes were examined at days 7, 14, 21, 28, 35, and 42. Mice groups 2 and 3 were immunized at days 0 and 15 by VP1-VLP vaccine and then challenged at day 22 by ip route successively with the diabetogenic CVB4E2 and with the wild-type CVB4JBV strains. Neutralizing antibody against E2 or JBV strains titers results are shown in Figure 3. Results showed that the antibody titers of all immunized groups except the PBS group (naive control mice) exhibited a significant increase progressively after vaccinations (prime and booster doses), peaking at day 35, that is 13 days after the challenge (Figure 3).

### 3.3. Serum IgG Antibodies Titers against VP1-VLP

**Figure 4:**
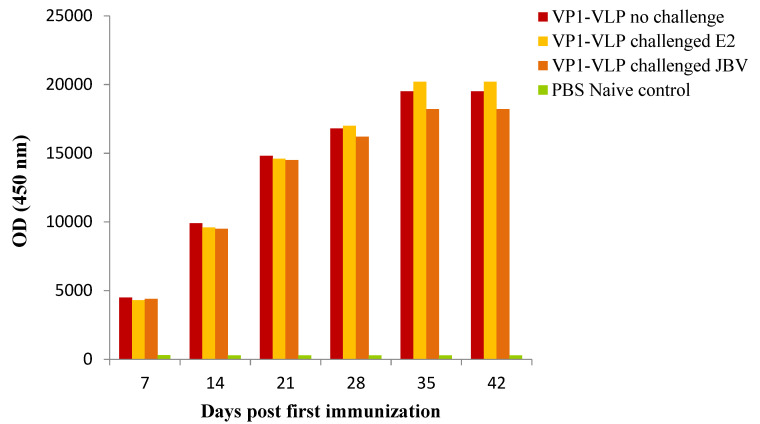
Antibody response. Serum IgG recognizing VP1-VLP in immunized mice. Titers of total IgG in the three immunized groups of mice. Mice (*n* = 5) receiving CVB4E2 VP1-VLP vaccine were immunized intraperitoneally by prime-boost regimen at days 0 and 15 with 0.5 μg of VP1-VLP. Mice were then challenged at day 22 with CVB4E2 or CVB4JBV pathogenic strains. PBS naïve mice group represents the control group. The results represent the mean of three replicate experiments.

The efficacy of the VP1-VLP vaccine was assessed by administration of a prime-boost regimen at days 0 and 15. Blood samples were collected at days 7, 14, 21, 28, 35, and 42 for assessment of IgG antibody responses to the VP1-VLP using ELISA method. All mice groups illustrated antigen-specific antibody responses (IgG) (Figure 4). The VP1-VLP vaccine elicited high titers at day 35 for all tested groups, demonstrating that the VP1-VLP particles are immunogenic. The mice group prime-boost immunized and challenged with E2 strain showed higher IgG responses compared to other groups. The naive control mice group demonstrated significantly very low titers compared to all other groups at different days post first immunization. Interestingly, during the observation of the IgG antibody titers against VP1-VLP, IgG titers from the E2 and JBV challenged groups did not show any significant difference (Figure 4), which indicates that VP1-VLP prepared from E2 immunization could cross-induce specific IgG antibodies against JBV, similar to the group challenged with E2 strain.

**Figure 5:**
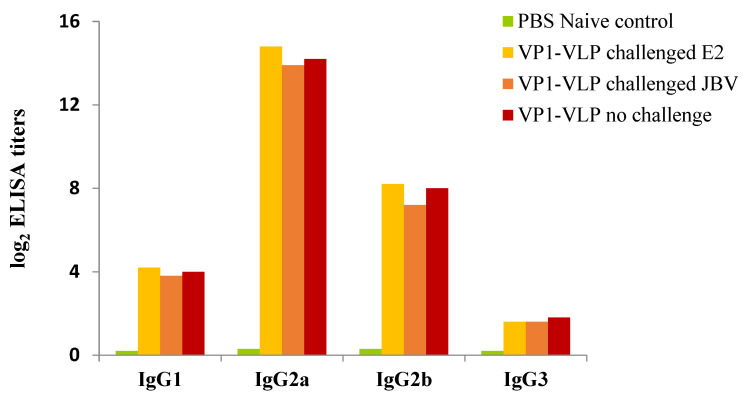
Antibody subtyping. Isotypes of the specific IgG antibodies in serum of the three VP1-VLP immunized groups. Sera from each mouse was obtained at day 35 post the first immunization and tested by ELISA method. PBS naïve mice group represents the control group.

Isotypes of IgG, IgG1, IgG2a, IgG2b, and IgG3 were evaluated in sera of mice groups collected at day 35, which represents the day with highest titers of IgG produced. IgG2a antibodies were elevated in all tested groups (Figure 5). Mice groups prime-boosted with the VP1-VLP vaccine and challenged or not challenged with E2 or JBV strains showed at day 35 post immunization an increased level of Th1 cells related subclass IgG2a compared to naive control mice group. In addition, the titers of all IgG antibody subtypes generated after challenge with either E2 or JBV strains had almost the same levels (Figure 5).

### 3.4. Cytokine Cellular Responses against VP1-VLP Vaccine

**Figure 6:**
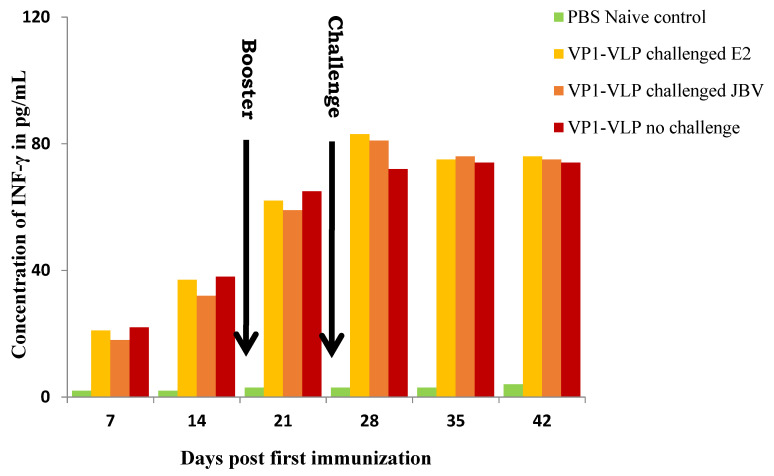
Measuring of IFN-γ by ELISA in the sera of the three different immunized groups. Sera of the mice group were collected at different days post the first immunization (as described in Section 2). The concentrations of IFN-γ were evaluated and measured by ELISA method. PBS naïve mice group represents the control group.

To detect the cellular immune responses in vaccinated mice groups, the concentrations of interferon gamma (IFN-γ) in sera of groups 1, 2, 3, and 6 were examined by ELISA method as described in Section 2 at days 7, 14, 21, 28, 35, and 42 post first immunization with VP1-VLP vaccine (Figure 6). The results showed that the interferon (IFN-γ) levels in the different immunization groups increased significantly compared to the naive control group (Figure 6), which demonstrated that VP1-VLP vaccine particles could effectively induce cellular immunity in mice.

### 3.5. Mice Protection from E2 and JBV Lethal Challenges by VP1-VLP Immunization

**Figure 7:**
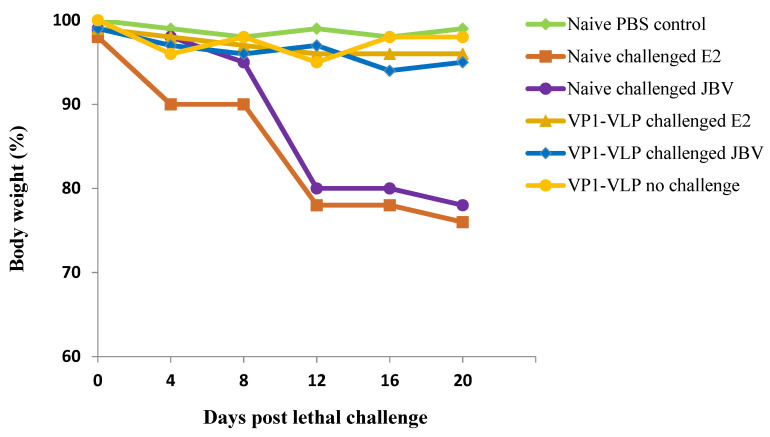
Body weight changes observed in all mice groups at different days post lethal challenge with pathogenic E2 and JBV strains. Mice groups not immunized and challenged by pathogenic strains showed substantial decrease in body weight percentage. PBS naïve mice group represents the control group.

**Figure 8:**
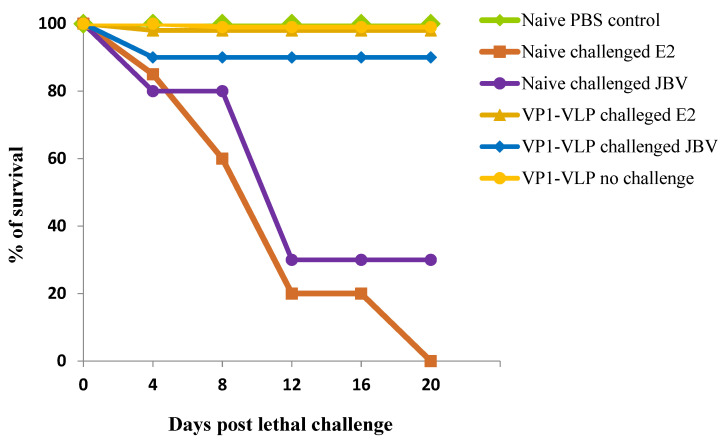
Survival percentage of all mice groups at different days after lethal challenge with pathogenic E2 and JBV strains. Mice groups not immunized and challenged by pathogenic strains showed the most elevated percentage of mortality. PBS naïve mice group represents the control group.

Mice groups 2, 3, 4, and 5 were challenged with diabetogenic E2 (1.1 × 10^6^ TCID_50_) or with wild-type JBV (1.36 × 10^6^ TCID_50_) strains as described in Section 2. However, mice group 1 was only immunized with VP1-VLP vaccine but not challenged. Mice group 6 represents naive control group mice receiving PBS with no challenge. Decrease in body weight was used as an indication of viral infection. Challenged mice were monitored daily for disease signs, and body weight changes for 20 days post challenge (Figure 7) were observed. All control naive mice challenged either with E2 or with JBV strains rapidly lost weight (more than 25%) and died or had to be euthanized at 20 days post challenge due to severe clinical symptoms. Mice groups receiving vaccine prime-boost with no challenge showed slight fluctuations less than 5% in body weight. However, no significant loss in body weight or clinical signs of infection was observed for naive PBS control group mice and for VP1-VLP immunized group mice with no challenge. The survival rates were different in the four challenged groups receiving the same lethal doses of E2 and JBV strains (Figure 8). Mice group challenged with the JBV strain dose of 1.36 × 10^6^ TICD_50_ and VP1-VLP-immunized showed a protective efficacy of 90%, whereas mice group challenged with the E2 strain dose of 1.1 × 10^6^ TICD_50_ and immunized with VP1-VLP showed a protective efficacy of 100%. The naive mice groups challenged with the same dose of JBV strain showed a protective efficacy of 30% at 20 days post challenge. However, naive mice group challenged with E2 strain showed no protective efficacy at 20 days post challenge, as all mice died at this stage. Naive PBS control mice group and VP1-VLP- immunized group with no challenge showed no significance mortality during the studied period post challenge.

### 3.6. Pancreas Tissue Viral Titers

**Figure 9:**
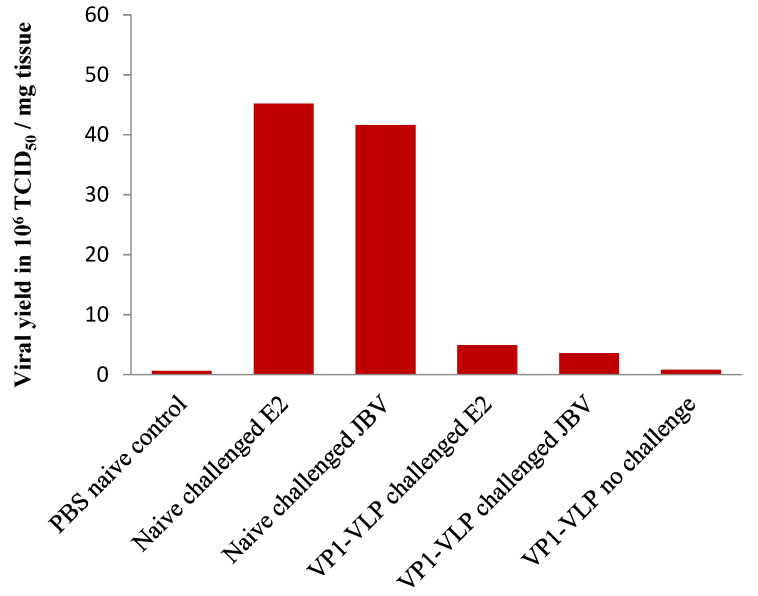
Viral load in the pancreas tissues of naive not challenged, naive challenged, and VP1-VLP vaccinated mice groups at day 20 after lethal CVB4E2 or CVB4JBV challenges (42 days post first immunization). Viral load (TCID_50_/mg) was determined in HeLa cells using Reed–Muench method [21]. VP1-VLP vaccinated and challenged mice groups showed a 10-fold lower viral load than the naive challenged mice groups.

Mice groups were sacrificed twenty days following lethal challenge with E2 or JBV strains and pancreas homogenates were prepared. HeLa epithelial cells were infected with pancreas samples and the viral titer was quantified by the tissue culture infectious doses in a given volume (TCID_50_). Figure 9 shows the viral yield in all the vaccinated mice and naive control mice groups. The viral load was shown to be 10-fold lower in the VP1-VLP vaccinated and challenged mice groups compared to the naive challenged group and to the VP1-VLP-immunized group not challenged (Figure 9). As expected, the viral load in the naive challenged mice groups was considerably high.

## 4. Discussion

The focus on vaccine production technologies has changed in the past decades from handling intact pathogens to producing recombinant subunit vaccines based on isolated target antigens [22]. Virus-like particles (VLPs) are virus-derived structures made up of one or more different molecules with the ability to self-assemble, mimicking the form and size of a virus particle but lacking the genetic material, so they are unable to infect the host cell. Expression and self-assembly of the viral structural proteins can take place in various living or cell-free expression systems after which the viral structures can be assembled and reconstructed. The systems used for the expression of individual heterologous proteins have been developed based on the prokaryotic and eukaryotic cells, which allow to produce target proteins both on laboratory and industrial scales [23]. VLPs are gaining in popularity in the field of preventive medicine, and to date, a wide range of VLP-based candidate vaccines have been developed for immunization against various infectious agents. VLPs are highly immunogenic and are able to elicit both the antibody- and cell-mediated immune responses by pathways different from those elicited by conventional inactivated viral vaccines [24,25]. The potential technological advantages of recombinant vaccines over conventional whole virus vaccines have led researchers to find suitable antigen proteins. Previously, we have produced and characterized VLPs containing the capsid protein VP1 self-assembling of CVB4E2 strain [20]. Capsid viral protein VP1 was selected as the vaccine antigen as it is highly conserved in the *Picornaviridae* family, the Enterovirus genus, and especially among CVB serotypes. We described the technology that allows the production of high-quality VP1-VLP for different applications. We have shown that the produced VP1-VLP was stable and highly purified by ultracentrifugation. The VP1-VLP was indistinguishable from the native virus in size, composition, and appearance, as determined by SDS–PAGE, Western blotting, and transmission electron microscopy.

Since the launch of polio vaccines in 1950s, only one enterovirus vaccine (against EV71) has been developed and registered in China for human use. One reason for the lack of enterovirus vaccines is due to the large number of different enterovirus serotypes (over 100), which circulate around the world and are rapidly and continuously recombining and evolving. It has not, therefore, been easy to identify causal serotype–disease relationships. There are, however, certain virus disease associations that are currently well established which make the development of novel vaccines worthy of consideration. Among such enteroviruses is CVB4, which causes severe infections leading to T1D [3,4,5,6,7]. Several vaccines against enterovirus 71 have been developed and studied in animal models, and human clinical trials have yielded promising results [26]. However, such success in the development of a CVB4 vaccine has not been made to date. In the present study, we demonstrate by vaccination experiments in mice models that produced VP1-VLP from the diabetogenic CVB4E2 strain is highly immunogenic, has high neutralizing titers, and provides protection against lethal challenges. We have explored the cross-protection between coxsackievirus B4 serotypes. The lethal challenge of two pathogenic strains, CVB4E2 and CVB4JBV, were used to determine the cross-protection induced by the VP1-VLP vaccine. The results showed that immunization with VP1-VLP could effectively induce specific neutralizing antibodies and protect mice against lethal dose of two pathogenic strains, E2 and JBV. The cross-protection between different coxsackievirus serotypes is very complicated; our results obtained with the most pathogenic CVB4 strains could be a lead for further investigations of cross-protection by expanding the population of coxsackievirus serotypes.

Interesting, the anti-VLP sera raised by VLP-VP1 vaccination exhibited a strong neutralizing capacity against both CVB4E2 and CVB4JBV strains. Titers of specific neutralizing antibodies increased progressively after prime-boost regimen and especially after challenge with pathogenic strains. In addition, we showed by ELISA that mice immunized with VLP-VLP had a strong serum IgG antibody response and that the most subclass was IgG2a, following by IgG2b. Our results of the specific antibody responses are very similar to many previous studies showing the same efficacy of enteroviral VLP as immunogens [27,28]. All of these studies measured immune responses by ELISA and/or seroneutralization as part of their main analyses. Specific neutralizing antibody titers were increased in this study by using a boost regimen at day 15 post immunization, reaching their maximum at day 35 post immunization. However, in many other studies, researchers used adjuvants to enhance the immune response and increase the specific neutralizing antibody titers. In a previous study, Koho et al. [29] were able to generate high neutralizing titers with smaller doses of purified VLP by chromatography, thus highlighting the purity and high immunogenicity of the chromatography purified VLPs. In the present study, VLPs were purified by ultracentrifugation; chromatography purification should enhance immunogenicity of our produced VLP vaccine.

We also examined the body weight changes and the survival rates of immunized and challenged mice groups. The results showed that the loss of total body weight was detected starting at 4 days post challenge in naïve mice groups challenged with E2 and JBV pathogenic strains. However, no significantly different body weight change was observed in VP1-VLP immunized and challenged groups compared with the naïve PBS immunized control group. When we examined the survival percentages of different mice groups, we revealed that the VP1-VLP immunized and E2 challenged mice group showed a protective efficacy of 100%, but only 90% of protective efficacy was revealed among VP1-VLP immunized and JBV challenged group. Mice groups not immunized and challenged by E2 and JBV strains showed the lowest survival rates compared to the PBS naïve control group. All these results indicated that VP1-VLP immunized mice could produce effective protection against both E2 and JBV pathogenic strains in mice models.

## 5. Conclusions

In conclusion, we present in this study the construction, production, and purification of an immunologically efficient and vaccine candidate for CVB4-related diseases in the form of VLP. Our present study illustrates that the produced VP1-VLP vaccine was able to induce a strong immune response in mice, as determined by seroneutralization and ELISA assays. It may play a role in protecting against the two most pathogenic CVB4E2 and CVB4JBV strains.
